# Objective Work-Related Factors, Job Satisfaction and Depression: An Empirical Study among Internal Migrants in China

**DOI:** 10.3390/healthcare8020163

**Published:** 2020-06-09

**Authors:** Nannan Zhang, Dinghong Chai

**Affiliations:** 1 Department of Social Work, School of Social and Public Administration, East China University of Science and Technology, Shanghai 200237, China; nnzhang@ecust.edu.cn; 2 Department of Social Work, School of Social Development, East China Normal University, Shanghai 200241, China

**Keywords:** objective work-related factors, job satisfaction, depression, migrants

## Abstract

This study examines the associations between objective work-related factors, job satisfaction and depression among migrants in China. Data from a representative sample of Chinese migrants named Management and Services of Migrants Study (MSMS) were used after excluding 1068 self-employed participants. We employed multivariate linear regression analysis. Depression was measured by the Centre for Epidemiologic Study Depression (C-ESD) scale. Objective work-related factors included firm size, job classification, mode of employment, working hours per week, union membership and working overtime compulsorily. Measurement of job satisfaction was derived from the Job Descriptive Index. We found that migrants in the sales/services sector and the clerical/technical/managerial sector had more depressive symptoms compared with those in the manufacturing/transportation sector. Working more than 55 h per week was associated with more depressive symptoms. Working overtime compulsorily and joining a labour union were all associated with more depressive symptoms. In addition, job satisfaction was negatively associated with depressive symptoms. The research findings on the relationship between work-related factors and depressive symptoms may serve as a guide for vocational rehabilitation counselling programs and for further research on depression in workplaces.

## 1. Introduction

Depression, a common psychiatric condition characterized by a depressed mood and reduced interest, has become a major concern worldwide [1,2]. This condition is the growing cause of disability associated with impaired quality of life and increased financial burden among working age populations [3].

Due to the economic imbalances between rural and urban areas, Chinese rural residents are attracted by the job opportunities in the cities and migrate to urban areas. They are the backbone of economic development [4]. These migrants, called internal migrants, move within the borders of a country, which results in a change in their general place of residence but not their household registration, or *hukou*. Under this household registration system, residents are categorized into urban (non-agricultural) and rural (agricultural) residents [4]. The *hukou* is viewed as an important contributor to migrants’ exclusion and marginalisation in China, as it plays an important role in resource allocation in healthcare, education and pension [5,6]. Chinese internal migrants are at high risk of experiencing depression due to acculturation stressors, such as discrimination, lack of social and financial resources, stress and unemployment-related frustration [7,8], and breaking ties with family [9]. The migrant population reached 244 million in 2017 in China [10]. Reports revealed that 23.7% of migrants in China exhibit significant depressive symptoms, and 12.8% are diagnosed as clinically depressed [11]. Migrants’ mental health problems pose challenges for policy practitioners and academic investigators. The Chinese migrant population has contributed extensively to the economic growth in recent decades [4]. Exploring mental health correlates of this population is of significant social and economic importance.

In order to better understand depression, an increasing number of empirical studies and theoretical perspectives focusing on correlates of depression have emerged. The causes of depression are manifested in variables such as income, health status [12], and education [13]. In addition to the studies regarding individual factors, research focusing on workplace factors has gained increasing attention in recent years [14,15,16]. Previous studies showed that work-related factors such as firm size [17], working hours [18,19], occupational grade [20], working environment and work stress [21], social relations at work [15,22], protection at work [16], psychological demands [23], and job satisfaction [24] are all significantly associated with depression. A study on American employed women suggested that working in a large firm and being a professional worker are protective factors for mental health [17]. Working hours were reported a risk factor for mental health [18,19]. A study among office staff aged 35–55 years from a London-based civil service department showed that employees working more than 55 h a week reported higher levels of depression compared with those who worked 35–40 h a week [18]. Another study conducted among Japanese first-year residents employed at training hospitals indicated that those who worked 80 or more hours per week showed significantly more depressive symptoms compared with those who worked less than 60 h a week [19]. Job satisfaction was proved as a protective factor for depression among professionals in the United States [24].

As Chinese migrants spend most of their time in the workplace, exploration of workplace effects on depression is of critical importance. Previous empirical studies regarding Chinese internal migrants’ depression were mainly on individual factors such as low monthly income, poor living condition, physical illness and having worked in many different cities [12]. Meanwhile, there are also a few explorative studies on the effects of work-related factors on depression. According to a qualitive study regarding migrants’ depression, migrants who experienced various forms of acculturative work stress report more depressive symptoms [7]. Working more than 8 h per day was also a risk factor for depression among rural-to-urban migrant workers in Shenzhen [12].

Accompanying with these changes, theoretical frameworks have also been provided to explain the workplace effects on depression. The culture-work-health model is one of these endeavours. This theoretical model focuses on objective work-related, psychosocial and organisational factors, and individual indicators. This model suggests that causes of individual mental health problems persist in individual character indicators (i.e., behaviour, perception or productivity) and work-related contextual factors (i.e., organisational management arrangement, structure and environment) [25]. Apart from these objective work-related factors, perceptions and responses to objective work-related factors are also closely linked to workplace mental health. All factors in the workplace constitute the culture of the organisation [25].

Despite the increasing number of studies on the correlates of migrant workers’ depression, most of these studies have been mainly on individual factors. The associations between objective work-related factors, satisfaction and depression among migrant workers are seldom discussed. The risk factors for depressive symptoms varies among populations with different social and cultural backgrounds, therefore, exploration of depression across different cultures are of critical importance.

We went beyond previous studies by including the objective work-related factors and the psychosocial factor, namely job satisfaction, into our analysis. Following the culture-work-health model, we provided the following hypotheses:

**Hypothesis** **1.** *Objective work-related factors were significantly positively associated with depression among internal migrants in China.*

**Hypothesis** **2.** *Job satisfaction was significantly negatively associated with internal migrants’ depression in China.*

## 2. Materials and Methods

The data used in this study were from the Management and Services of Migrants Study (MSMS), which was conducted in 2013. It has been seven years since the data were collected. Despite the time elapsed, migrants are still classified as temporary residents under the *hukou* system. In addition, migrants are still confronted with work-related risk factors at workplaces [12]. In 2013, 35.8% of migrants were working in the manufacturing industry [26]. Although this figure decreased to 27.9% in 2019, the proportion of migrant population working in the manufacturing industry is still larger compared with the proportion of migrants in other industries [27].

The sample size of MSMS accounted for 0.0016% of the migrant population, which reached 245 million in 2013. To be eligible for the study, respondents had to satisfy the following criteria: (a) they had lived in the hosting city for more than one month; (b) they were aged above 16 years old; (c) their *hukous* were not in the hosting cities and (d) they knew the purpose of the study and voluntarily gave written consent to participate. Purposive sampling and convenience sampling methods were combined in determining the sample. In brief, we first purposively selected eight cities based on the geographic location and economic development. These cities were situated in the eastern (Shanghai, Tianjin, Guangzhou, Sanya), middle (Wuhan, Haerbin) and western (Chengdu, Lanzhou) parts of China. These cities are representative of different geographical regions and various levels of social and economic development in China. Second, a total of 500 participants in each city were selected under the snowball sampling method. As migrant workers have high mobility and are unregistered in the Urban Hukou Management System, obtaining a sampling frame for random sampling among migrant workers became impossible [7]. Therefore, the convenience sampling method was used in the second stage. Finally, 3801 eligible participants were invited to participate in this survey. The response rate was 95%. Based on the question of the mode of employment (regular employment vs self-employed), we finally had 2733 migrants working in factories after excluding 1068 self-employed participants, as the self-employed migrants lacked a stable working environment. The final sample size of this study was 2733. All participants provided written informed consent prior to participating in the survey.

### 2.1. Analytical Strategy

Descriptive statistics were used to establish mean values and frequencies of variables. Spearman correlation analyses were applied to examine the interrelationships of the independent and confounding variables with depression. Linear regression analyses were conducted to determine the associations between objective work-related factors, job satisfaction and depression. Demographic factors including age, gender, marital status, income, and education were controlled as confounding variables. Significant level was set at *p* < 0.05 for all analyses. All analyses were conducted using STATA 14 (Stata Corporation, College Station, TX, USA).

### 2.2. Measurement

#### 2.2.1. Depression

Depression was measured through the seven-item Centre for Epidemiologic Studies Depression (CES-D) scale [28], which is the short version of the original 20-item CES-D. Previous studies showed that CES-D exhibited good reliability and validity among the Chinese population [29,30]. This instrument is widely used among the migrant population in China [31]. This scale proved good reliability among this migrant population, and the Cronbach’s α equals to 0.839 in this sample. This scale included seven negatively described items, such as, (a) I felt that everything I did was an effort; and (b) I felt stressed. Items were scored on a four-point Likert-scale that ranged from 0 (rarely or none) to 3 (most or a little of the time). Higher scores indicated more depressive symptoms.

#### 2.2.2. Objective Work-Related Factors

The objective work-related factors included firm size, job classification, mode of employment, working hours per week, union membership and working overtime compulsorily. We followed a definition in a previous study to measure firm size [17]. The firm size was treated as a dichotomous variable. Companies with 1000 or more employees were considered large firms, whereas companies with less than 1000 employees were categorized as small firms. The measurements of job classification and mode of employment followed the measures used in a previous study among working women in a Japanese metropolis [32]. Job classification included three categories—namely, manufacturing/transportation employees, sales/service staff and clerical/technical/managerial employees. Employees in the same category (i.e., clerical, technical, and managerial employees) shared the relatively similar objective work-related factors. Each category was considered a dummy variable with manufacturing/transportation as the reference category. Mode of employment included two categories, namely, government institution staff and others. More specifically, government institution staff referred to those who worked in the government sectors, while others consisted of part-time, contract and dispatched employees in the non-governmental sectors.

There was no consensus on how to define the long working hours. We followed the research on British civil servants [18], which categorised the participants into three groups, namely, 1 = less than 40 h/week, 2 = 41–55 h/week and 3 = more than 55 h/week. We recoded working hours into a set of dummy variables in the analysis, wherein the reference category was less than 40 h/week.

Compulsory overtime work was considered a dummy variable with those who were not forced to work compulsorily as the reference group. Labour union membership was measured by a dummy variable, where the reference group included migrants without union membership.

#### 2.2.3. Job Satisfaction

The measurement of job satisfaction was derived from the Job Descriptive Index (JDI) and it has good reliability and validity among the Chinese population [33]. Job satisfaction comprised satisfaction levels of nine subscales, such as salary, working environment, and time arrangement. These items were measured through 4-point Likert scales ranging from very dissatisfied (1) to very satisfied (4). Job satisfaction was the summation of these items, ranging from 9 to 36. Higher scores reflected higher levels of job satisfaction.

#### 2.2.4. Confounding Variables

The confounding variables in this research included age, gender, marital status, income and education. Age was treated in the continuous form. Gender and marital status were measured by two dummy variables. Income was measured in the log transformed income. Education was measured by a continuous variable ranging from 1 (never attend any school) to 8 (graduate school and above).

## 3. Results

Among all the depressive symptoms, the highest mean score was the item “I always feel stressed” (Mean = 1.042, SE = 0.751). The lowest mean score was the item “I feel life is meaningless” (Mean = 0.355, SE = 0.597). In this sample, only 9.448% (*N* = 262) of the participants had never been affected by any of the depressive symptoms. Table 1 presents the descriptive statistics and reveals that all participants were relatively young, with an average age of approximately 30 years.

The number of male migrant workers was slightly higher than that of the female migrant workers (58.5% vs 41.46%). Among the participants, 44.16% were married. The average yearly income of the participants was RMB 77,653.56 (about USD 11,000). More than 50% of the participants received high school and higher education. Regarding the objective work-related factors, 84.11% of the migrant participants worked in small firms, 11.64% of the participants were employed as sales/service staff, and 43.1% of the participants were engaged in clerical/technical/managerial jobs. In this sample, 7.43% of the respondents worked in government institutions. Migrants who worked for more than 55 h a week accounted for 35.18% of all participants. Among the respondents, 17.64% of them reported their experience of compulsorily overtime work. In addition, 12.77% of the migrants were labour union members. The mean score of aggregated satisfaction was 24.64 (SD = 3.96).

Table 2 reflects the Spearman correlation of the independent and confounding variables with depression. The rough analysis reveals that firm size (*p* < 0.05), clerical/technical/managerial (*p* < 0,01), government institutions staff (*p* < 0.05), compulsory overtime work (*p* < 0.001), union membership (*p* < 0.05) and job satisfaction (*p* < 0.001) were all significantly associated with depression among migrants. In the following multivariate regression analyses, these variables were included into the models to examine their associations with depression by controlling for the confounding variables.

Table 3 illustrates the association between objective working factors and depression. In Model A, we found that older migrants (b = −0.35, *p* < 0.001) who were married (b = −0.453, *p* < 0.01) reported significantly fewer depressive symptoms than younger unmarried migrants. We further entered the objective work-related factors into Model B (Table 3). In Model B, we found that older individuals significantly had fewer depressive symptoms (b = −0.030, SE = 0.008). Married (b = −0.421, SE = −0.299) male migrant workers had fewer depressive symptoms compared with female unmarried migrant workers. The objective work-related factors alone accounted for 5% of the variance in depressive symptoms. In Model C, we further entered income and education on the basis of Model B. 

In Model C (Table 3), compared with migrants who worked in the manufacturing/transportation sectors, migrants who worked in the sales/service sector (b = 0.459, *p* < 0.05) and the clerical/technical/managerial sectors (b = 0.314, *p* < 0.01) were expected to have more depressive symptoms. Migrants who worked for more than 55 h a week had 0.581 more units in depression compared with those who worked for less than 40 h a week (b = 0.581, *p* < 0.01). However, we did not find significant differences in depressive symptoms between migrants who working for less than 40 h a week and those working for 40 to 55 h a week. Migrants who worked overtime compulsorily reported 1.772 more units in depression (b = 1.722, *p* < 0.001) than those who were not compelled to work overtime. Migrants participating in the labour union had 0.457 more units in depression (b = 0.457, *p* < 0.05) compared with those who were non-union members. Working in a large firm or a small firm did not make a significant difference on migrants’ depressive symptoms. Together with the confounding variables, all the objective work-related factors accounted for 9% of the variance in depression. In addition, we found that working overtime compulsorily was the most damaging objective work-related factor among all the objective work-related factors.

Table 4 shows the association between job satisfaction and depressive symptoms (Model A and Model B) and the associations among objective work-related factors, job satisfaction and depressive symptoms (Model C). In Model A, we found that every one-unit increase in job satisfaction was associated with 0.188 units decrease in depression (b = −0.188, *p* < 0.001). In Model B, we found that job satisfaction and confounding factors collectively accounted for 9% of the change in depressive symptoms among migrants. In Model C, we entered all the objective work-related factors and job satisfaction into the model and found that these factors could explain 11% of the variance in depressive symptoms.

## 4. Discussion

We examined objective work-related factors and job satisfaction as predictive factors of depressive symptoms in a sample of migrant workers in China. To the best of our knowledge, this is the first systematic study on the associations between objective work-related factors, job satisfaction and depression among Chinese migrants. Our findings provide selective support for Hypothesis 1 which suggested that objective work-related factors were positively significantly associated with depression. We found that migrants in the sales/services sector and the clerical/technical/managerial sector had more depressive symptoms compared with those in the manufacturing/transportation sector. Working more than 55 h per week is associated with more depressive symptoms. Working overtime compulsorily and joining labour union were all associated with more depressive symptoms. In addition, job satisfaction was negatively associated with depressive symptoms. Our Hypothesis 2, which suggested a negative association between job satisfaction and depression, was supported. 

Working in a large firm was not a significant predictive factor of depressive symptoms among the migrant population in China, which is in line with an earlier cross-sectional study which showed that working in a large firm did not significantly affect depression among working women [17]. Migrants who were in the sales/service and clerical/technical/managerial sectors exhibited more depressive symptoms than those who were in labour occupations (i.e., manufacturing, transportation). This finding is inconsistent with a previous finding among U.S. workers showing no significant differences in depression among different occupations [34]. Migrant workers working in the sales/service and clerical/technical/managerial sectors have more opportunities to communicate with people compared with those in the manufacturing/transportation. This fact might partially explain that migrant workers in sales/service and clerical/technical/managerial occupations reported more depressive symptoms compared with those in manufacturing/transportation occupations. 

Migrants who worked for more than 55 h per week reported significantly more depressive symptoms, whereas migrants who worked for 40 to 55 h per week did not exhibit significantly more depressive symptoms compared with those working for 40 h or less per week. Our results are partially consistent with a previous study showing that working more than 55 h/week predicted subsequent depressive symptoms among men, while working 41–55 h/week was a predictive factor for mental health among women [18]. Our findings indicate that moderately long working hours (40–55) are not significant predictors for depression among migrant workers. This might reflect the fact that moderate overtime work has higher levels of resilience in a society with rapid social and economic development (i.e., China). In addition, qualities like diligence are cherished in traditional Chinese values, which may also drive migrants to accept moderate overtime work. Furthermore, migrants who were compelled to work overtime exhibited more depressive symptoms than those who were not. In fact, compulsorily working overtime is common among numerous firms. In our sample, 17% of the respondents reported compulsory overtime work. Compulsory overtime work is associated with low decision latitude and high demand latitude among migrant workers [35], which may further exacerbate depression.

Being a labour union member reflected increased depressive symptoms in the current study. A previous study suggested that labour union membership is positively correlated with job satisfaction and self-rated health [36]. The relationship between labour union membership and depression may be explained by the fact that labour unions in companies in China failed to function well for improving the psychological well-being of migrant workers. In addition, migrants with labour union membership may hold higher expectation for improvement regarding benefits and working environment, therefore they are more sensitive to these factors. When a large gap exists between these expectations and the reality, there is an increased risk for labour union members to be depressed. The association between job satisfaction and depression is consistent with the study conducted among U.S. professionals [24]. Job satisfaction is a general subjective evaluation of objective working factors and work arrangement. Job satisfaction serves as a protective factor for depression or a modulator to ease the negative effects of risk indicators on mental health. 

Our study has practical implications. This research provided empirical evidence for practitioners. Improving migrants’ working environment is a protective measure for their mental health. Specifically, compulsory overtime work increased the risk of depression. Therefore, measures protecting migrants from mental health risks could be considered in management. Furthermore, a previous study showed that flextime actually improves productivity and reduces absenteeism [37]. Although implementing flextime in some industries is unrealistic, delivering the autonomy in deciding whether to work overtime to employees is possibly effective in preventing employees from developing depressive symptoms. Additionally, policy makers could take measures to improve migrant workers’ mental health through building a positive subjective working environment. 

Findings from the current study could potentially be harnessed to deepen understanding on the vocational rehabilitation counselling and human resource management in the Chinese context. Clinicians could guide the migrant workers to handle the stressors well or change their work settings to deal with their depression. By examining work-related factors, human resource managers can easily identify vulnerable employees for targeted intervention. Enterprises could benefit economically by promoting the psychological wellbeing of employees, as healthy workers showed improved efficiency and enhanced productivity [38,39]. 

## 5. Conclusions

This study has identified several objective work-related factors and job satisfaction as being related to the depression of migrant workers in China, which was rarely discussed in the literature. This study found that some objective work-related factors and job satisfaction were all significantly associated with depressive symptoms among migrant workers in China. In addition, this empirical study has implications for human resource management in companies. Protective measures focusing on improving migrant workers’ working environment and job satisfaction should be taken.

Despite the above contributions, this study has several limitations. First, we merely examined the association between work-related factors and depression and failed to investigate the causal effects between these factors due to the data constraints. Second, this study examined the objective work-related factors and job satisfaction among migrant workers in China, and might not be generalizable to migrant populations in other countries. Therefore, organisational health and longitudinal studies should be considered in the future.

## Figures and Tables

**Table 1:** Descriptive statistics (*N* = 2733).

Variables	Mean	SD	Percent	Number	Range
Age	29.71	9.86			15–67
Gender					
Male			58.54%	1600	
Female			41.46%	1133	
Marital status					
Married			44.16%	1207	
Others			55.84%	1526	
Income	77,653.56	67,957.43			1600–999,999
Education					
Never attend any school			0.95%	26	
Primary school			7.61%	208	
Middle school			25.39%	694	
High school			13.65%	373	
Occupational school			8.85%	242	
Junior college			18.26%	499	
College			21.59%	590	
Graduate school and above			3.70%	101	
Firm size					
Large			15.89%	428	
Small			84.11%	2266	
Job classification					
Manufacturing/transportation			45.26%	1237	
Sales/services			11.64%	318	
Clerical/technical/managerial			43.10%	1178	
Mode of employment					
Government institutions staff			7.43%	203	
Others			92.57%	2530	
Working hours					
Less than 40 h/week			33.27%	908	
40–55 h/week			31.55%	861	
More than 55 h/week			35.18%	960	
Compulsory overtime work			17.64%	482	
Non-compulsory overtime work			82.36%	2251	
Union member			12.77%	349	
Non-union member			87.23%	2384	
Job satisfaction	24.64	3.96			9–36
CES-D	4.71	3.23			0–21

Note: SD = standard deviation.

**Table 2:** Spearman correlation of independent and confounding variables with depression.

Variables	Depression
Age	−0.156 ***
Male	−0.037
Married	−0.140
Income (ln(income))	−0.049 **
Education	0.128 ***
Large firm	0.038 *
Sales/services	0.043 *
Clerical/technical/managerial	0.056 **
Government institutions staff	0.041 *
More than 40 h/week	0.002
More than 55 h/week	0.001
Work overtime compulsorily	0.215 ***
Union member	0.403 *
Job satisfaction	−0.224 ***

Note: * *p* < 0.05, ** *p* < 0.01, *** *p* < 0.001.

**Table 3:** Association between objective work-related factors and depression.

Variables	Model A		Model B		Model C	
	b	β	b	β	b	β
Age	−0.035 *** (0.008)	−0.107	−0.030 *** (0.008)	−0.991	−0.029 *** (0.008)	−0.089
Male	−0.181 (0.125)	−0.028	−0.299 * (0.124)	−0.045	−0.291 * (0.124)	−0.044
Marital status	−0.453 ** (0.164)	−0.070	−0.421 * (0.164)	−0.065	−0.299 (0.166)	−0.46
Income (ln(income))					−0.319 *** (0.082)	−0.073
Education					0.125 ** (0.047)	0.070
Firm size			0.136 (0.168)	0.015	0.107 (0.168)	0.012
Sales/services			0.486 * (0.204)	0.048	0.459 * (0.208)	0.046
Clerical/technical/managerial			0.396 ** (0.139)	0.061	0.314 * (0.152)	0.048
Government institutions staff			0.289 (0.235)	0.023	0.204 (0.237)	0.017
More than 40 h/week			0.194 (0.155)	0.028	0.243 (0.157)	0.035
More than 55 h/week			0.457 ** (0.163)	0.067	0.581 ** (0.175)	0.086
Work overtime compulsorily			1.771 *** (0.159)	0.208	1.722 *** (0.159)	0.203
Union member			0.490 ** (0.188)	0.051	0.457 * (0.188)	0.047
Adjusted *R*^2^	0.03		0.08		0.09	
Number of observations	2733		2691		2691	

*Note*: Standardize errors were in the parentheses. * *p* < 0.05, ** *p* < 0.01, *** *p* < 0.001.

**Table 4:** Association between job satisfaction, objective work-related factors and depression.

Variables	Model A		Model B		Model C	
	b	β	b	β	b	β
Age	−0.037 *** (0.008)	−0.112	−0.030 *** (0.008)	−0.093	−0.028 ** (0.008)	−0.086
Male	−0.229 (0.121)	−0.035	−0.219 (0.121)	−0.033	−0.294 * (0.122)	−0.447
Marital status	−0.464 ** (0.159)	−0.71	−0.246 (0.163)	−0.038	−0.268 (0.163)	−0.041
Income (ln(income))			−0.251 ** (0.082)	−0.058	−0.271 ** (0.082)	−0.062
Education			0.199 *** (0.037)	0.113	0.134 ** (0.047)	0.075
Firm size					0.146 (0.165)	0.016
Sales or services					0.650 ** (0.206)	0.065
Clerical/technical/managerial					0.441 ** (0.150)	0.067
Government institutions staff					0.118 (0.233)	0.010
More than 40 h/week					0.134 (0.155)	0.019
More than 55 h/week					0.377 * (0.173)	0.556
Work overtime compulsorily					1.267 *** (0.163)	0.149
Union member					0.437 * (0.185)	0.045
Job satisfaction	−0.188 *** (0.015)	−0.230	−0.193 *** (0.015)	−0.236	−0.157 *** (0.016)	−0.191
Adjusted *R*^2^	0.08		0.09		0.11	
Number of observations	2733		2733		2691	

*Note*: Standardize errors were in the parentheses. * *p* < 0.05, ** *p* < 0.01, *** *p* < 0.001.
